# Atrial-His bundle pacing in fulminant myocarditis with ventricular arrhythmia: a case report

**DOI:** 10.1186/s12872-022-02936-8

**Published:** 2022-11-22

**Authors:** Limeng Jiang, Liangguo Wang, Chuhuan Zhao, Xi Zhou, Xia Hong, Xiafei Feng, Lei Xu, Shengjie Wu, Roy Chung, Weijian Huang, Lan Su

**Affiliations:** 1 Department of Cardiology, The First Affiliated Hospital of Wenzhou Medical University, Nanbaixiang, Wenzhou 325000 P.R China; 2 The Key Lab of Cardiovascular Disease of Wenzhou, Wenzhou, China; 3 Department of Cardiac Intensive Care Unit, The First Affiliated Hospital of Wenzhou Medical University, Wenzhou, China; 4 Heart and Vascular Institute, Cleveland Clinic, Cleveland, OH USA

**Keywords:** His bundle pacing, Atrioventricular block, Fulminant myocarditis, Echocardiography, Case report

## Abstract

**Background:**

Fulminant myocarditis is a clinical syndrome associated with threatening dysrhythmia which temporary pacemaker can be used for life-saving support. As a method of physiological pacing, His bundle pacing (HBP) maintain better cardiac synchronization than traditional right ventricular (RV) pacing.

**Case presentation:**

It’s a severe case of fulminant myocarditis in a 41-year-old patient who presented for recurrent arrhythmias with hemodynamic instability. Temporary His bundle pacing combined with optimal medical therapy and extracorporeal membrane oxygenators (ECMO) supported him through his critical period of hospitalization.

**Conclusions:**

During 1-year follow up, the cardiac function recovery was obvious without any pacing related complications. Echocardiography showed better atrioventricular and intra-ventricular synchronization during HBP in DDD mode. This is the first reported case of temporary His-purkinje conduction system pacing used for severe fulminant myocarditis.

## Background

Fulminant myocarditis is a clinical syndrome with severe symptoms of acute heart failure, cardiogenic shock, or life-threating rhythm disturbances which should be treated with full supportive care, using aggressive pharmacologic therapy and mechanical circulatory support [1]. His-purkinje conduction system pacing as a physiologic pacing method can achieve cardiac resynchronization which is similar to native conduction. We report a unique case of severe fulminant myocarditis with recurrent malignant ventricular arrhythmias, complete heart block and hemodynamic instability. Atrial-His bundle pacing supported the patient hemodynamics through this critical period of his hospitalization.

## Case presentation

**Fig. 1:**
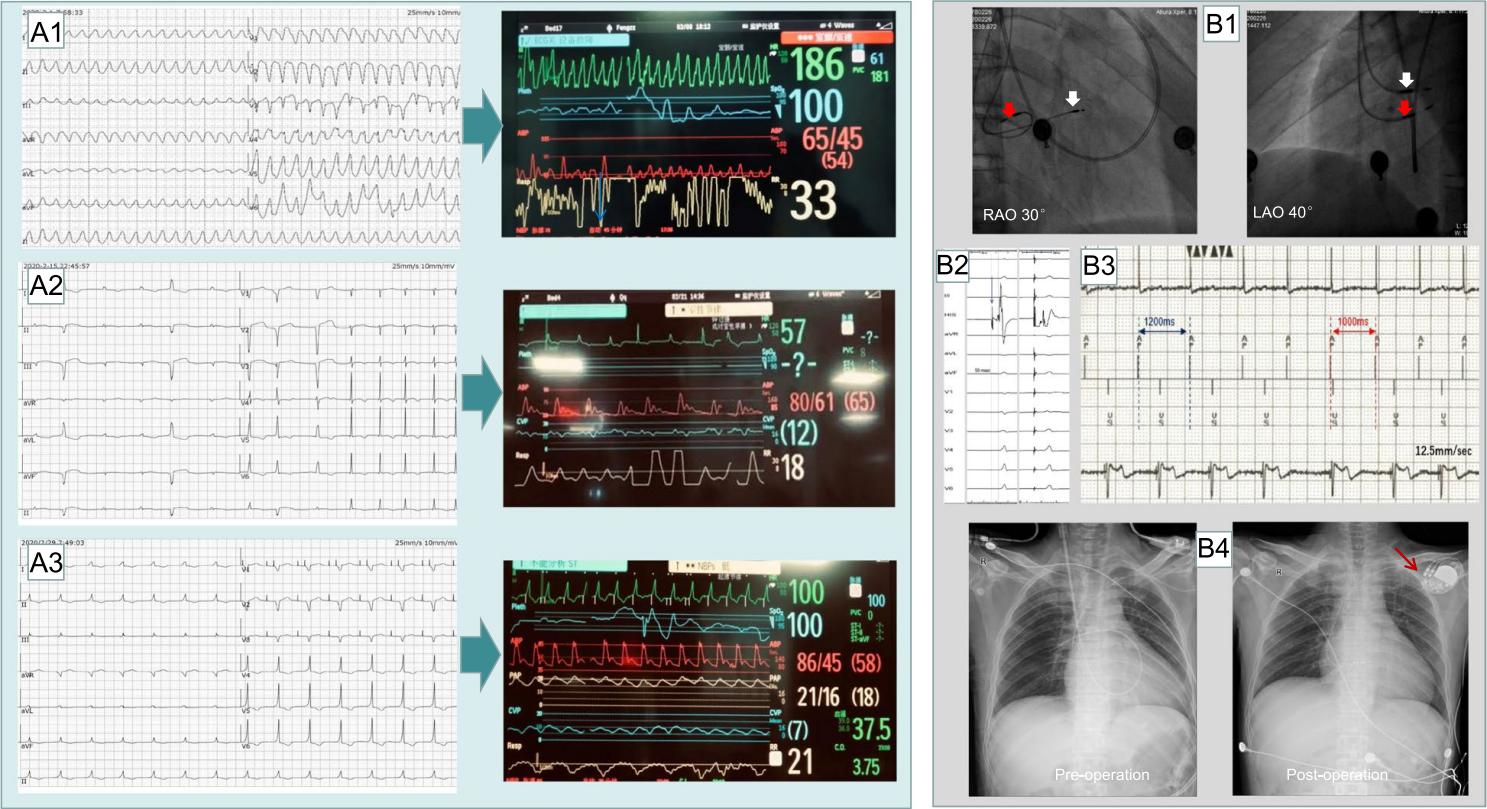
Electrocardiogram, hemodynamic monitoring and radiographs. **A1**: ECG showed ventricular tachycardia, corresponding to hemodynamic instability before temporary His bundle pacing. The paper speed was 25 mm/s; **A2**: ECG showed sinus arrest with ventricular escape rhythm and central venous pressure of 12 mmHg before operation. The paper speed was 25 mm/s; **A3**: ECG showed atrial-ventricular sequential pacing in DDD pacing mode with hemodynamics improved postoperatively. The paper speed was 25 mm/s; **B1**: Fluoroscopic RAO 30°and LAO 40° projection showed relative position of the pacing lead in the low right atrium (red thick arrow) and His bundle (white thick arrow) position respectively; **B2**: Intra-cardiac electrogram showed big His potential (blue arrow) with HV interval of 59 ms and selective HBP at 1 V/0.5 ms. The paper speed was 100 mm/s; **B3**: Intra-cardiac electrogram recorded on pacemaker programmer showed atrial pacing at 50 bpm could realize 1:1 atrial to ventricle conduction, while atrial pacing at 60 bpm resulted Wenckebach phenomenon of atrioventricular conduction. The paper speed was 12.5 mm/s; B4: Chest radiographs showed pulmonary congestion and cardiothoracic ratio increased to 0.63 before temporary conduction system pacing (left) while reduced to 0.59 after device implantation (right), the red arrow showed that the temporary pacemaker was fixed on the skin

A 41-year-old previously healthy male presented to our hospital after a week of fever. He had dyspnea and hypotension. He had no long-term medication. He did not smoke cigarettes, take alcohol or illicit drugs. Hypoxic on presentation, he required supplemental oxygen via nasal cannula. Heart rhythm was irregular but he did not have any murmur. Laboratory data on admission showed an alanine aminotransferase (ALT) level of 3904 U/L, creatine kinase (CK) level of 2463 U/L, creatine kinase-MB (CK-MB) level of 193 U/L, troponin I (cTnI) level of 42.38 ug/l, brain natriuretic peptide level of 734 pg/ml, and serum creatinine level of 111umol/l. The laboratory test of herpesvirus IgM antibody showed positive and the result of metagenome and target gene sequencing was negative. Chest radiograph showed obvious pulmonary congestion (Fig. 1 B4). Twelve lead electrocardiogram (ECG) during hospitalization demonstrated ventricular tachycardia (Fig. 1 A1) and sinus arrest with ventricular escape rhythm (Fig. 1 A2). Intra-cardiac electrogram recorded on pacemaker programmer showed atrial pacing at 60 bpm resulted Wenckebach phenomenon of atrioventricular conduction (Fig. 1 B3). Coronary angiography revealed no coronary artery stenosis and left ventriculography showed diffuse hypokinesis. Left ventricular ejection fraction (LVEF) by echocardiography demonstrated mean ejection fraction of 25%.

Diagnosis of fulminant myocarditis might have different underlying causes and pathogenetic processes-viral, bacterial, toxic, and autoreactive. Histological and immune-histological specimens made from the biopsies help diagnosis in the clinical course. However, in this case heart failure and severe arrhythmias dominated the clinical manifestations which made myocardial biopsy impossible. Eventually, from the history, clinical examination and electrocardiogram, fulminant myocarditis associated with cardiogenic shock was our primary diagnosis. Initially, norepinephrine, dobutamine, milrinone and other positive inotropic drugs and vasoactive agents were used to maintain his vital signs. However, cardiogenic shock and arrhythmias could not be corrected, then ECMO was used in hospital day 11. Despite the contemporary use of ECMO for three weeks, cardiogenic shock persisted with conduction disease, associated with recurrent monomorphic ventricular tachycardia, requiring sequential atrioventricular pacing support (Fig. 1 A). Due to the concern of cardiac dys-synchronization caused by conventional temporary RV pacing, we performed HBP to prevent further deterioration of cardiogenic shock and the inability to wean him off mechanical circulatory support. Conventional two-chamber pacemaker was selected for temporary pacing device. A lead delivery system consisting of C315 His catheter (Medtronic Inc., Minneapolis, MN, USA) and Select Secure 3830 pacing lead (Medtronic Inc., Minneapolis, MN) was placed at the septal area of the atrioventricular junction. In that region endocardial mapping was performed using the lead tip until a clear His bundle potential was obtained with HV interval of 59 ms (Fig. 1 B2). At this level, pacing at variable amplitudes showed nonselective His bundle capture with important narrowing of the paced QRS complex. Selective HBP was achieved at 1.25 V/0.5 ms with R wave of 3.6mv and bipolar impedance of 430Ω. HBP at 140 bpm resulted in 1:1 HV conduction. Besides, in addition to sinus arrest, the patient also had problems with atrioventricular conduction, therefore a dual chamber DDD pacing mode was programmed to preserve atrioventricular activation as part of the most physiological conduction rather than single chamber AAI or VVI pacing mode. The total procedural time was 53 min, and no procedural-related complication was noted in a fully anticoagulated state with heparin due to the presence of ECMO.

Postoperative pacing rate was adjusted to 80-100 bpm, with invasive hemodynamic monitoring by arterial line and right cardiac catheterization demonstrating optimal pulmonary arterial pressure (PAP), cardiac output (CO), cardiac index (CI), central venous pressure (CVP) and invasive arterial pressure (IAP) (Table 1). The pacing parameters were stable. Remarkably, his ventricular tachycardia ceased within 24 h of HBP. ECMO was decannulated 8 days after pacemaker implantation. Considering the lack of sinoatrial node and atrioventricular node recovery two weeks after temporary HBP, permanent pacemaker was implanted using the same venous access. We selected HBP combined with back-up right ventricular septal pacing (RVSP) to prevent the increase of His capture threshold and useless of His electrode due to the progression of conduction disease to distal position. During his follow-up at 1 year, selective His bundle captured threshold and non-selective His bundle captured threshold at 1-year follow-up were 0.5 V/0.5 ms and 1.5 V/0.5 ms, respectively. Echocardiography reported a remarkable improvement of LVEF to 58%, along with optimal heart failure medical therapy (Table 1). 12-lead ECG showed sinus arrest and atrioventricular block with the Wenckebach block point of AV conduction maintained at 60 bpm.

**Table 1:** Invasive hemodynamic monitoring and echocardiographic parameters

	Invasive hemodynamic monitoring					Echocardiographic parameters				Recommendations and Interventions
	PAP (mmHg)	CVP (mmHg)	CO (L/min)	CI (L/min.m2)	IAP (mmHg)	LVEDD (mm)	LVEF (%)	MR	TR	
Before temporary HBP	15/10	12	3.02	1.95	74/39	52	22	mild	mild or moderate	-Persistent ECMO for more than 10 days
The first day post temporary HBP	18/9	12	3.79	2.34	91/43	48	43	mild	moderate	-ECMO -Temporary HBP with DDD mode
8 days post temporary HBP	16/9	8	4.17	2.63	109/50	47	43	mild	moderate	-ECMO was removed -Temporary HBP with DDD mode
The first day post permanent HBP	-	-	-	-	-	47	44	mild	moderate	-Permanent atrial-HBP which maintains the better atrial-Hisian-ventricular
1 month post permanent HBP	-	-	-	-	-	49	45	mild	moderate	
1 year post permanent HBP	-	-	-	-	-	49	58	normal or mild	mild or moderate	

*Abbreviations*: *PAP* Pulmonary arterial pressure, *CVP* Central venous pressure, *CO* Cardiac output, *CI* Cardiac index, *IAP* Invasive arterial pressure, *LVED* Left ventricular end diastolic dimension, *LVEF* Left ventricular ejection fraction, *MR* Mitral regurgitation, *TR* Tricuspid regurgitation

**Fig. 2:**
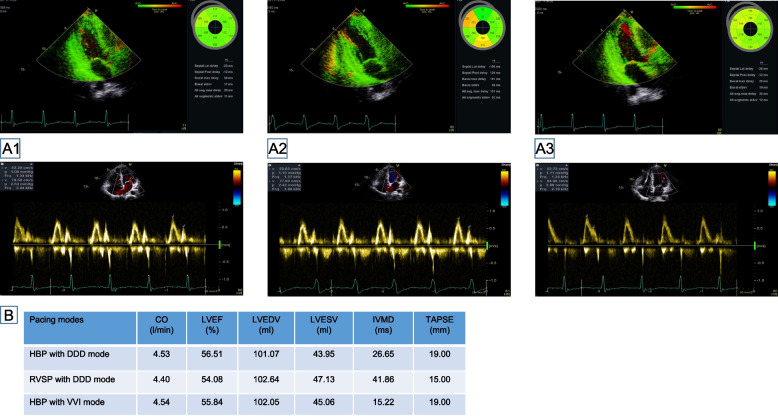
Echocardiography at 1-year follow-up. Echocardiography at 1-year follow-up showed cardiac mechanical synchrony and ventricular systolic function in three different pacing modes. **A1**: HBP with DDD mode; **A2**: RVSP with DDD mode; **A3**: HBP with VVI mode. **B**: RVSP exhibited a greater intraventricular mechanical delay (IVMD) compared with HBP pacing. HBP with DDD mode showed optimum atrioventricular synchrony and left ventricular systolic function. HBP: His bundle pacing; RVSP: Right ventricular septal pacing; CO: Cardiac output; LVEF: Left ventricular ejection fraction; LVEDV: Left ventricular end-diastolic volume; LVESV: Left ventricular end-systolic volume; APEI: Aortic pre-ejection time; PPEI: Pulmonary pre-ejection time; IVMD: Intraventricular mechanical delay; TAPSE: Tricuspid annular plane systolic excursion

The cardiac mechanical synchrony was separately evaluated by transthoracic echocardiography (TTE) in three different pacing modes with HBP in DDD mode, RVSP in DDD mode and HBP in VVI mode at 1-year follow-up, with a washout period of 5 min between each pacing mode. In terms of left ventricular function, HBP with DDD mode showed better left ventricular (LV) synchrony evaluated by tissue Doppler imaging (TDI) and LV systolic function reflected by LVEF and CO than RVSP. RV systolic function with HBP evaluated by tricuspid annular plane systolic excursion (TAPSE) was better than that of RVSP. Finally, the intraventricular mechanical delay (IVMD) which was defined as the difference between the LV pre-ejection period (aortic pre-ejection time, APEI) and RV pre-ejection period (pulmonary pre-ejection time, PPEI) was used to evaluate mechanical delay between the left and right ventricle, showed a significant difference between HBP and RVSP. RVSP had a worse IVMD compared to HBP. In addition, DDD pacing mode showed optimum atrioventricular synchronization with better morphology of mitral peak early diastolic velocity (E’), mitral peak late diastolic velocity (A’) and mitral E/A ratio (Fig. 2).

## Discussion and conclusions

Fulminant myocarditis is a clinical syndrome characterized by severe myocardial inflammation frequently associated with threatening dysrhythmia manifested as bradycardia, conduction block, ventricular tachycardia and ventricular fibrillation. ECMO use is commonly required for fulminant myocarditis patients with hemodynamic instability to support them through the acute phase of cardiogenic shock [2, 3]. Conduction system injury involving sinoatrial node and atrioventricular junction was persistent in this patient which mainly manifested with uncorrectable sinus arrest and AVB requiring temporary pacing as a mean for chronotropic support [4]. However, traditional RV endocardial pacing in the setting of cardiogenic shock may lead to further deterioration of hemodynamics due to worsening LV function. Therefore, two 3830 active-fixation leads were selectively fixed in the low septum of right atrium and His bundle position with DDD pacing mode of atrial-Hisian-ventricular sequential conduction to achieve the precise and entire atrioventricular and intra-ventricular synchronization. Consequently, with the use of the most physiological pacing mode and ECMO, this patient had the most optimal clinical outcomes through temporary conduction system activation and mechanical circulatory support for hemodynamic collapse.

The effectiveness [5, 6] and safety [7–9] of conduction system pacing have been confirmed, however there are no report of conduction system pacing used for temporary pacing for fulminant myocarditis. This case illustrates an alternate approach that in patients with fulminant myocarditis, especially those with conduction disease and heart failure, His purkinje conduction system pacing with DDD mode may be preferable to conventional temporary right ventricular apical pacing which maintains the better atrial-Hisian-ventricular synchronization. Finally, clinical studies in experienced centers in the future can provide more evidence for this approach.

## Patient Perspective

“My heart failure symptoms resolved after optimal medical therapy and pacemaker implantation. My functional capacity returned to baseline at 1 year follow up. I’m tremendously grateful to the conduction system pacing team at the First affiliated hospital of Wenzhou university.” The patient talked about it at 1-year follow-up.

## Data Availability

All the data supporting our findings are contained within the manuscript.

## Abbreviations

HBP: His bundle pacing
RV: Right ventricular
ECMO: Extracorporeal membrane oxygenators
ALT: Alanine aminotransferase
CK: Creatine kinase
CK-MB: Creatine kinase-MB
cTnI: Troponin I
AVB: Atrioventricular block
LVEF: Left ventricular ejection fraction
PAP: Pulmonary arterial pressure
CO: Cardiac output
CI: Cardiac index
CVP: Central venous pressure
IAP: Invasive arterial pressure
RVSP: Right ventricular septal pacing
TTE: Transthoracic echocardiography
LV: Left ventricular
TDI: Tissue Doppler imaging
TAPSE: Tricuspid annular plane systolic excursion
IVMD: Intraventricular mechanical delay
APEI: Aortic pre-ejection time
PPEI: Pulmonary pre-ejection time
E’: Mitral peak early diastolic velocity
A’: Mitral peak late diastolic velocity

## References

[1] Ginsberg F, Parrillo J (2013). Fulminant myocarditis. Crit Care Clin.

[2] Kim I, Yang H, Kim W (2015). Pathological substratum for a case of fulminant myocarditis treated with extracorporeal membrane oxygenation and subsequent heart transplantation. J Korean Med Sci.

[3] Liu D, Xu J, Yu X (2019). Successful treatment of fulminant myocarditis in an adult in emergency department: A case report. Medicine (Baltimore).

[4] Adachi Y, Kinoshita O, Hatano M (2017). Successful bridge to recovery in fulminant myocarditis using a biventricular assist device: a case report. J Med Case Reports.

[5] Su L, Wu S, Wang S (2019). Pacing parameters and success rates of permanent His-bundle pacing in patients with narrow QRS: a single-centre experience. Europace.

[6] Su L, Wang S, Wu S, et al. Long-Term Safety and Feasibility of Left Bundle Branch Pacing in a Large Single-Center Study. Circ Arrhythm Electrophysiol. 2021;14(2):e009261. 10.1161/CIRCEP.120.009261 33426907

[7] Su L, Xu T, Cai M (2020). Electrophysiological characteristics and clinical values of left bundle branch current of injury in left bundle branch pacing. J Cardiovasc Electrophysiol.

[8] Upadhyay GA, Cherian T, Shatz DY (2019). Intracardiac Delineation of Septal Conduction in Left Bundle-Branch Block Patterns. Circ.

[9] Beer D, Subzposh FA, Colburn S (2021). His bundle pacing capture threshold stability during long-term follow-up and correlation with lead slack. Europace.

